# Effect of turmeric extract on bone healing in an experimental model of femoral bone fracture

**DOI:** 10.22038/AJP.2021.18561

**Published:** 2022

**Authors:** Shahab Ilka, Afshin Heshmati, Seyed Alireza Mirabdollahi, Abdollah Jafarzadeh, Farnaz Sedghy, Fatemeh Bagheri, Omid Azari, Mohammad Ali Mohammadi, Fatemeh Jafari Dareh Dar, Moein Arabnadvi

**Affiliations:** 1 *Department of Orthopedics, School of Medicine, Kerman University of Medical Sciences, Kerman, Iran*; 2 *Department of Immunology, School of Medicine, Kerman University of Medical Sciences, Kerman, Iran*; 3 *Pathology and Stem Cell Research Center, Pathology Department, School of Medicine, Kerman University of Medical Sciences, Kerman, Iran*; 4 *Department of Clinical Sciences, Faculty of Veterinary Medicine, Shahid Bahonar University of Kerman, Kerman, Iran*; 5 *Department of Parasitology, School of Medicine, Kerman University of Medical Sciences, Kerman, Iran*; 6 *Department of Cardiology, School of Medicine, Kerman University of Medical Sciences, Kerman, Iran*

**Keywords:** Femur fracture, Bone healing, Turmeric extract, Curcumin, Experimental model

## Abstract

**Objective:**

Following bone trauma, several factors participate in making a balance between the activity of osteoblasts and osteoclasts. The receptor activator of nuclear factor kappa B ligand (RANKL), receptor activator of nuclear factor kappa B (RANK), and osteoprotegerin (OPG) molecules play critical roles in the healing process via regulation of osteoclasts function. Turmeric is suggested to have an anti-osteogenic potential; however, its effect on accelerating bone healing has not been adequately studied. Here, we used a rat model of femur fracture to explore the effect of treatment with turmeric extract on the bone repair and the expression of *RANK*, *RANKL*, and *OPG* molecules.

**Materials and Methods:**

Eight rats were subjected to surgery, randomly divided into two groups, and treated orally with turmeric (200 mg/kg), or olive oil. Four oil-treated rats without bone fracture were used as control group. After six weeks of treatment, the femurs of animals were examined for radiological, histological, and gene expression analysis.

**Results:**

X-ray radiography showed thicker callus and a more obscure fracture line in the turmeric group. Furthermore, higher osteoblast percentages but no osteoclasts were observed in turmeric-treated animals, representing better repair of bone in the fracture site. Also, real-time analyses showed that treatment with turmeric reduced *RANK* and *RANKL* expression (p<0.0001) and lowered *RANKL*/*OPG* ratio (p=0.01) in femoral bone tissue.

**Conclusion:**

Our findings indicated the turmeric ability to facilitate bone hemostasis and optimize the expression of key markers involved in the bone metabolism.

## Introduction

Bone homeostasis depends on a balance between bone resorption and formation processes mediated by osteoclasts and osteoblasts, respectively (Mahmoudi et al., 2012; Lee et al., 2018; Chaugule et al., 2019; Koohpeyma1et al., 2020). After bone trauma, the healing process is mediated by various soluble factors as well as the cell to cell interactions. In this context, the receptor activator of nuclear factor kappa B ligand (RANKL), receptor activator of nuclear factor kappa B (RANK), and osteoprotegerin (OPG) are mainly involved in the regulation of bone metabolism (Chaugule et al., 2019). In the initial stage, inflammatory response leads to the differentiation of mesenchymal stem cells to osteoblasts, which produce RANKL, a powerful inducer of osteoclasts differentiation and activation via its interaction with RANK, expressed on the surface of osteoclast precursors (Guerrini and Takayanagi, 2014; Chaugule et al., 2019; Ferreira-Junior et al., 2020). OPG, another soluble osteoblast receptor with antagonistic activity against RANKL, can prevent RANKL-RANK interaction (Lacey et al., 1998; Histing et al., 2012). Therefore, the RANKL to OPG ratio (RANKL/OPG) determines the status of osteoclast function and bone metabolism (Gravallese et al., 2000; Abdallah et al., 2005; Lee et al., 2018). 

Despite the strong and regulated healing ability of the bone, 5-10% of bone fractures cannot be healed, leading to impaired quality of life and significant health care cost (Li et al., 2018; Safali et al., 2019). Curcumin is the active component of turmeric (*Curcuma longa*), with a variety of therapeutic properties (Tamaddonfard et al., 2012; Li et al., 2018). Potent antioxidant and anti-inflammatory activities of this medicinal plant, indicated in many previous studies (Arshami et al., 2012; Li et al., 2012; Yu et al., 2012; He et al., 2015; Zhang et al., 2016; Wang et al., 2017; Zhao et al., 2017; Kadam et al., 2018; Yang et al., 2018; Safali et al., 2019; Yasbolaghi Sharahi et L., 2020), might positively affect the healing process (Alaseirlis et al., 2005; Park et al., 2010; Güleç et al., 2018; Ferreira-Junior et al., 2020). Currently, some evidence in the literature supports the role of curcumin in the bone remodeling process, by its negative effect on RANKL and inhibiting osteoclastogenesis (Chen et al., 2012; Moon et al., 2012; Rohanizadeh et al., 2016). However, to our knowledge, few studies have investigated the anti-osteoclastogenic effect of turmeric or its active components on the healing process in fracture-induced animal models. In this research, we aimed to evaluate the role of turmeric extract on the expression of *RANK*, *RANKL*, and *OPG* using a Wistar rat model of femoral bone fracture.

## Materials and Methods

**Animals, groups, and doses**

The experimental protocol was carried out in accordance with the National Institutes of Health guidelines on animal care. Twelve male Wistar rats (*Rattus norvegicus*) of 12-14-week-old, weighing 175−230 g were maintained under 12 hr light and dark cycles (temperature 20−25°C) and fed on a standard diet with water *ad libitum*. Eight rats were subjected to surgery, randomly divided into two groups, and treated orally with turmeric (200 mg/kg body weight for six weeks after surgical procedure), or olive oil. Four normal rats without bone fracture were used as a healthy control group and treated with the same volume of olive oil. Animals were anesthetized and sacrificed 24 hr after the last administration of turmeric extract or olive oil (on day 42 after surgical procedure) and their femur underwent radiological, histological, and gene expression analysis.

All procedures were performed according to the National Institute of Health guidelines for the use of experimental animals to minimize animal suffering and approved by Animal Ethics committee in animal use of Kerman University of Medical sciences (protocol number: 9800121).

**Preparation of turmeric extract**

To prepare turmeric extract, dried rhizome of *Curcuma longa* L. was purchased from Iran (Kerman) and 100 g was homogenized in 95% ethanol for 24 hr at room temperature under reflux. The crude extract was filtered, and concentrated in a rotary evaporator at 60°C.

**Surgical procedure**

Rats were anesthetized by injection of xylazine (25 mg/kg, intraperitoneal) and ketamine (75 mg/kg, intraperitoneal). The patella was dislocated by a medial parapatellar incision at the right knee. A 0.5-mm diameter hole was drilled into the intercondylar notch and a needle (0.4 mm in diameter) was inserted into the intramedullary canal. The needle was removed after insertion of a tungsten guide wire (0.2 mm in diameter) through the needle into the intramedullary canal. The femur was fractured by a 3-point bending device and the fracture was stabilized using an intramedullary titanium screw (18 mm in length and 0.5 mm in diameter). Finally, the wound was closed using 6-0 synthetic sutures. Fracture induction was confirmed by radiography.

**Radiographic analysis**

After the induction of bone fracture, the Anterior-Posterior (AP) axis of animals was radiographed by a digital radiograph X-ray machine. The primary reduction of the fracture site was evaluated by the Kirschner wire (K wire). In both groups, fractures were transverse or oblique and non-comminuted with displacement less than 1 mm. X-ray radiography was repeated at the end of the second and sixth weeks of study. 

**Histologic analysis**

The femoral fracture sections were analyzed by an experienced pathologist in a blinded manner, using hematoxylin and eosin (H&E) staining. The sections were fixed in 10% formaldehyde for 24 hr and decalcified using 10% hydrochloric acid (HCl). The samples were then dehydrated using a gradient of ethanol solutions followed by paraffin embedding. Finally, sections of 3-μm thickness were prepared and stained with H&E, and examined under a light microscope (magnification, 1000x). The analysis was performed based for the following parameters: presence or absence of inﬂammation, granulation tissue formation, abscess formation, neovascularization, osteoblast and osteoclast percentages, and skin repair. To quantify the results, 10 high power fields (HPF) were counted and the percentage of each parameter was determined as the ratio of the parameter per 10 HPF.

**Quantitative real-time PCR**

Total RNA was extracted from femoral bone tissue using TRIzol reagent (YTA, Yekta Tajhiz Azma, Iran) and reverse transcribed to cDNA using cDNA synthesis kit (YTA, Yekta Tajhiz Azma, Iran) according to the manufacturer's instructions. For assessment of relative gene expression, the cDNA was amplified using SYBR green PCR master mix (YTA, Yekta Tajhiz Azma, Iran) for 45 cycles of 5 sec denaturation at 95°C, 20 sec annealing at 60°C, and 25 sec of amplification at 72°C using a Rotor-Gene Q instrument (Rotor Gene-Q, Qiagen, Germany). The expression levels of target genes were normalized to that of the housekeeping gene *GAPDH*. The primer sequences used for PCR analysis were designed using Beacon Designer 7 software and are listed in Table 1. Analysis of relative gene expression was performed using the Livack method (Livak and Schmittgen 2001). All experiments were performed in triplicate.

**Table 1:** Primers used in the real-time PCR assay

**Target gene**	**Primer sequence (5'-- 3')**	**product length (bp)**
*OPG*	Forward: GCAACCGCACCCACAACC	169
	Reverse: TCACCTGAGAAGAACCCATCCG	
*RANK*	Forward: CACACCAAGGGACGACGGAATC	133
	Reverse: GCCACCACTACCACAGAGATGAAG	
*RANKL*	Forward: CATCGCTCTGTTCCTGTACTTTCG	144
	Reverse: GCTTCTGTGTCTTCGCTCTCC	
*GAPDH*	Forward: AGTTCAACGGCACAGTCAAGGC	122
	Reverse: GACATACTCAGCACCAGCATCACC	

**Statistical analysis**

All data are presented as means± standard deviations (SD). After testing for normality by Kolmogorov-Smirnov test, Student’s t-test or one-way ANOVA (or their nonparametric equivalents) with Tukey’s *post hoc* test was used to compare the differences between experimental groups. Statistics were performed using Graph-Pad Prism 5.0 software (Graphpad, San Diego, CA) and p-values <0.05 were considered statistically significant. 

## Results

**X-ray imaging showed promoted fracture healing in turmeric-treated animals**

Six weeks after the induction of bone defect in Wistar rat model, X-ray investigations showed that the callus at the fracture site of control untreated group was thinner and the fracture line was more clearly visible, whereas the callus of the turmeric-treated group was thick and the fracture line was blurred on imaging, indicating better bone healing (Figure 1, supplementary Figure 1).

**Histological examination showed improved healing process in femoral fracture in rats treated with turmeric extract**

Histological examination was performed after six weeks of follow up (Table 2). The percentage of osteoblasts was higher in the turmeric-treated group compared to the control untreated animals. In contrast, osteoclast percentages were higher in the control group, while no osteoclast was observed in turmeric-treated animals. Moreover, after six weeks of treatment, tissue granulation and neovascularization were substantially higher in turmeric-treated animals. No considerable difference was observed in cellular infiltration between the two groups. No abscess was formed in the groups (Figure 2, supplementary Figure 2).

**Turmeric extract reduced** ***RANK*** **,** ***RANKL*** **, and** ***OPG*** **expression in rats fracture model**

Real-time PCR analysis demonstrated that the expression of *RANK*, a marker on the surface of osteoclasts, was significantly reduced after treatment with turmeric extract (p<0.0001, Figure 3a, supplementary Table 1-3). Also, the expression of *RANKL*, an essential factor for osteoclast differentiation and activation, and inducing bone resorption, was significantly decreased in the turmeric-treated group compared with the olive oil-treated group (p<0.0001, Figure 3b, supplementary Table 1-3). Moreover, the expression of *OPG*, which has antagonistic activity against *RANKL* and inhibits osteoclastogenesis, was reduced by turmeric treatment (p<0.0001, Figure 3c, supplementary Table 1-3).

**Table 2:** Tissue parameters in fracture site after six weeks of treatment with turmeric extract

**Tissue parameter (%)**	**Olive oil-treated group**	**Turmeric-treated animals**
Tissue granulation	53	90
Neovascularization	33	72
Osteoblasts	10	25
Osteoclasts	5	0

**Figure 1:**
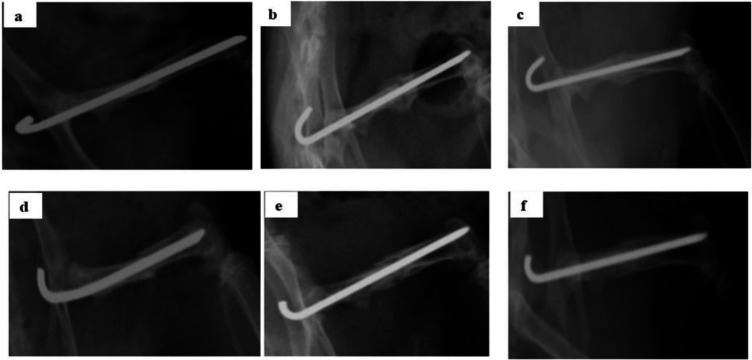
X-ray radiography of the fracture healing on the day of surgery (day 0) (a and d), and at the second week (b and e), and sixth week (c and f) in Wistar rats showed that turmeric extract promotes the fracture healing. The callus at the fracture site of the turmeric-treated group (c) was thicker and the fracture line was blurred on imaging, whereas the callus of the olive oil-treated group (f) was thin and the fracture line was more clearly visible, indicating better bone healing after treatment with turmeric extract

**Figure 2:**
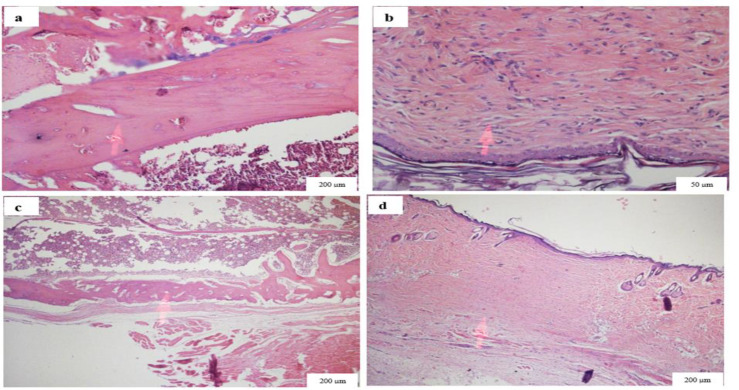
Histological analysis of femur fracture site six weeks post-surgery. Hematoxylin and eosin staining of the fracture site in the turmeric–treated animals (a and b) and the olive oil-treated group (c and d) showed that the percentage of osteoblasts was higher in the turmeric-treated group, while osteoclasts percentage was higher in the control group. Moreover, tissue granulation and neovascularization were notably higher in the turmeric-treated animals. Cellular infiltration was observed in both groups. No abscess was formed in any studied animals (Hematoxylin and eosin staining, 200×)

**Figure 3:**
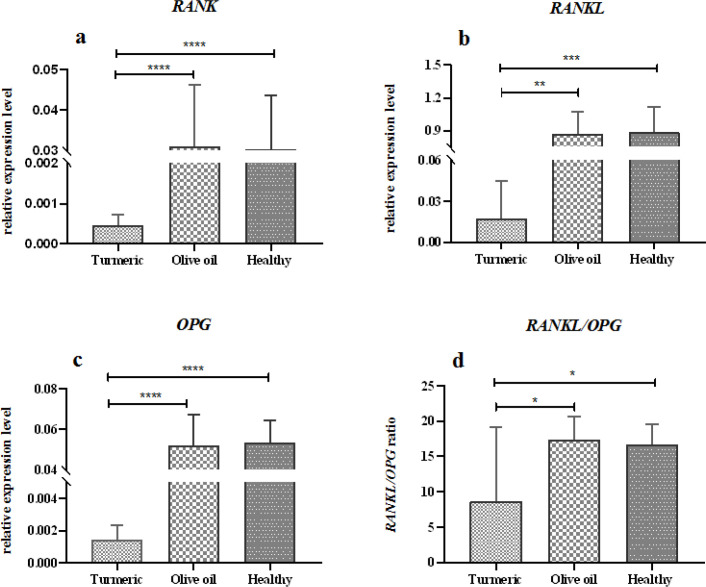
Relative expression of *RANK*, *RANKL*, and *OPG* in turmeric-treated compared to the control animals in the femoral fracture-induced rat model after six weeks of treatment. Relative expression of *RANK* (a), *RANKL* (b), and *OPG* (c) was significantly decreased after turmeric treatment (p<0.0001). Also, The *RANKL*/*OPG* ratio (d) was significantly lower in the turmeric-treated group, but higher in the both control (olive oil-treated and healthy) animals (p=0.01)

**Turmeric extract decreased the** ***RANKL*** **/** ***OPG*** **ratio in the rat model of fracture**

The effect of six weeks of treatment with turmeric extract on the *RANKL*/*OPG* ratio in this rat model of femoral fracture was determined. The *RANKL*/*OPG* ratio was 5 in the turmeric-treated group, but it was significantly higher in the control untreated animals (p=0.01, Figure 3d, supplementary Table 1-3), indicating a dramatic decrease in the level of *RANKL* compared to *OPG* in the turmeric-treated animals.

## Discussion

The potential of turmeric extract and its active components in promoting the healing process has been reported in some recent studies (Yang et al., 2015; Li et al., 2018; Safali et al., 2019). In this work, we explored the effect of six weeks of treatment with turmeric extract on the bone healing process using a rat model of femur fracture. Our gene expression analyses showed that turmeric extract promoted fracture healing by affecting the expression of bone remodeling markers. Turmeric treatment reduced *RANK* and *RANKL* expression in the femoral bone of rats. Interaction between RANK and RANKL is necessary for activation and function of osteoclasts, resulting in bone resorption in the remodeling process (Chen et al., 2016). We showed that administration of turmeric extract decreased the expression of the two markers and therefore, alleviated the bone resorptive activity of osteoclast. These results are consistent with previous research indicating the ability of curcumin to limit activation of osteoclasts via decreasing *RANKL* expression in bone marrow stromal cells and RAW264.7 cells (Oh et al., 2008; Park et al., 2011). Some evidence suggested that pro-inflammatory cytokines promoted the binding of RANKL to RANK, leading to the activation of osteoclastic signaling pathways and their differentiation and maturation (Kang et al., 2016; Yang et al., 2016; Yang et al., 2019); considering the anti-inflammatory properties of curcumin demonstrated in many research (Chainani-Wu 2003; Menon and Sudheer 2007; Ak and Gülçin 2008; Basnet and Skalko-Basnet 2011; Hewlings and Kalman 2017; Kadam et al., 2018; Safali et al., 2019), downregulation of these genes was in accordance with our expectations. OPG and RANKL are pivotal factors in the bone repair process and increased RANKL/OPG ratio was considered a marker of bone resorption (Chen et al., 2016). Cirano et al. (2018) reported that curcumin treatment abates the ratio of RANKL/OPG in calvarial samples of rats with diabetes mellitus; we consistently found that *RANKL*/*OPG* ratio was markedly lowered in femoral bone tissue of turmeric-treated animals. These data showed the potential of turmeric to promote the balance between the expressions of the two markers, and accordingly, bone protective properties that might be attributed to this treatment. 

Histological and radiological evaluation revealed that turmeric facilitated bone healing in this Wistar rat model. We assessed the callus diameter using X-ray radiography. Radiologic evaluation showed thicker callus; moreover, fracture line was obscure in the turmeric group after six weeks of treatment, representing that bone healing was better at the fracture site. In support of these findings, Li et al. (2018) explored the effects of curcumin on femoral fracture repair by comparing callus density in treated and untreated rats using X-ray and micro-computed tomography (CT) and they showed similar results. However, another study (Safali et al., 2019) that measured the ratio of the callus diameter to intact bone diameter on imaging analysis, found no significant difference in the diameter of the callus between the turmeric and the control group; this may be due to the shorter (four weeks) follow-up time. 

Also, we showed higher percentages of osteoblasts in the turmeric-treated group after six weeks of administration of turmeric extract. In contrast, no osteoclast was observed in the treated animals. As expected, increased tissue granulation and neovascularization were shown in treated animals. In a previous research by Cheng et al. (2017), curcumin was able to improve glucocorticoid-induced osteoporosis by inhibiting apoptosis in osteoblasts. Another group reported that curcumin could increase the number of osteoblasts and protect against bone loss in an ovariectomized rat model (Hussan et al., 2012). Also, Hie et al. (2009) analyzed blood and femoral bone data from rats with insulin-dependent diabetes mellitus and reported a decline in the bone resorption and osteoclast activity after receiving a dietary supplement of curcumin.

There are also several limitations to our study. First of all, higher numbers of animals should be used for more valid results. Moreover, we analyzed the data after six weeks of administration of turmeric; it would be better to collect the data at various time points for better interpretation of results. 

Taking together, our findings indicated the ability of turmeric to optimize the expression of critical markers involved in the bone healing. Hence, further research should be done on the effects of curcumin on osteoclasts and osteoblasts, the main effector cells in bone remodeling, to elucidate the mechanisms by which curcumin mediates these effects.

## Conflicts of interest

The authors have declared that there is no conflict of interest.

## Supplementary

**Figure 1:**
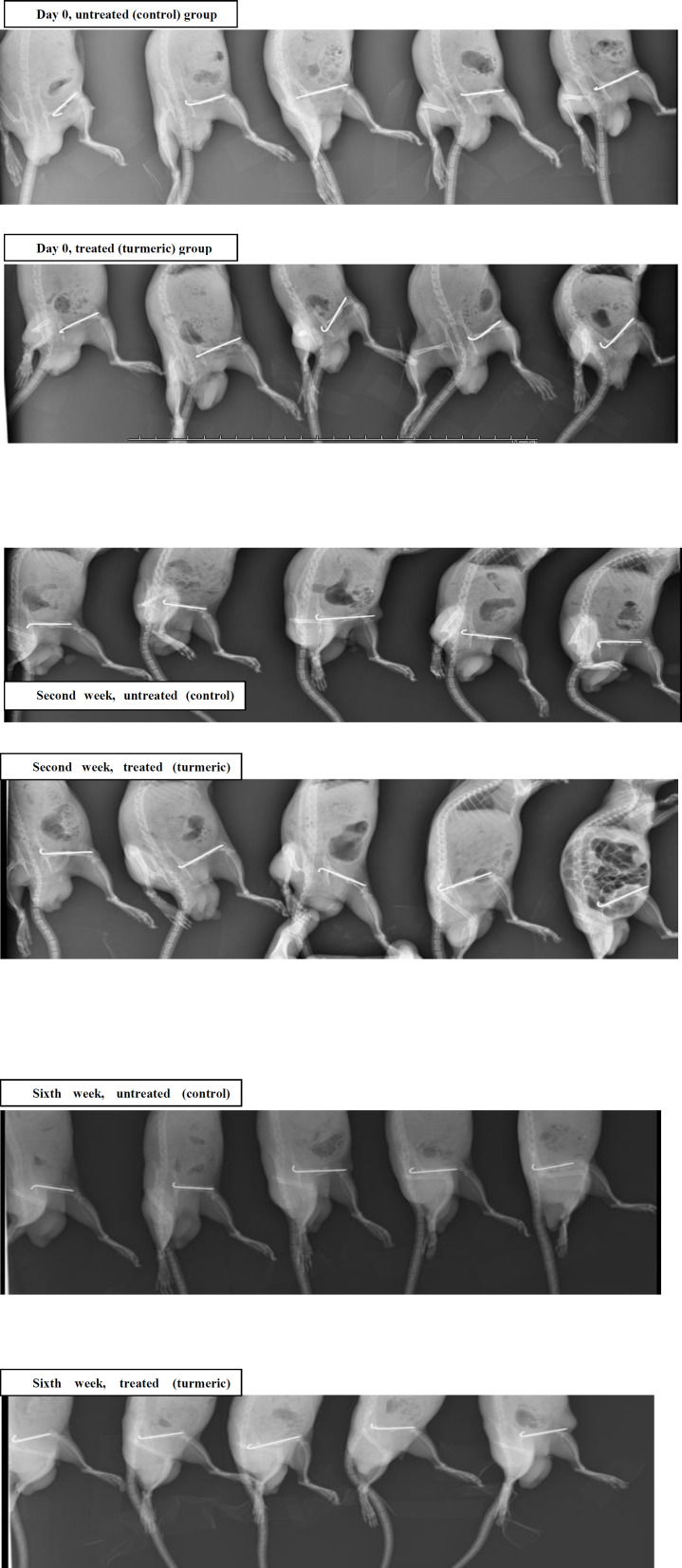
X-ray radiography at the day of surgery, at the second week**, **and sixth week of treatment of Wistar rat model showed that turmeric extract promotes the fracture healing.

**Figure 2:**
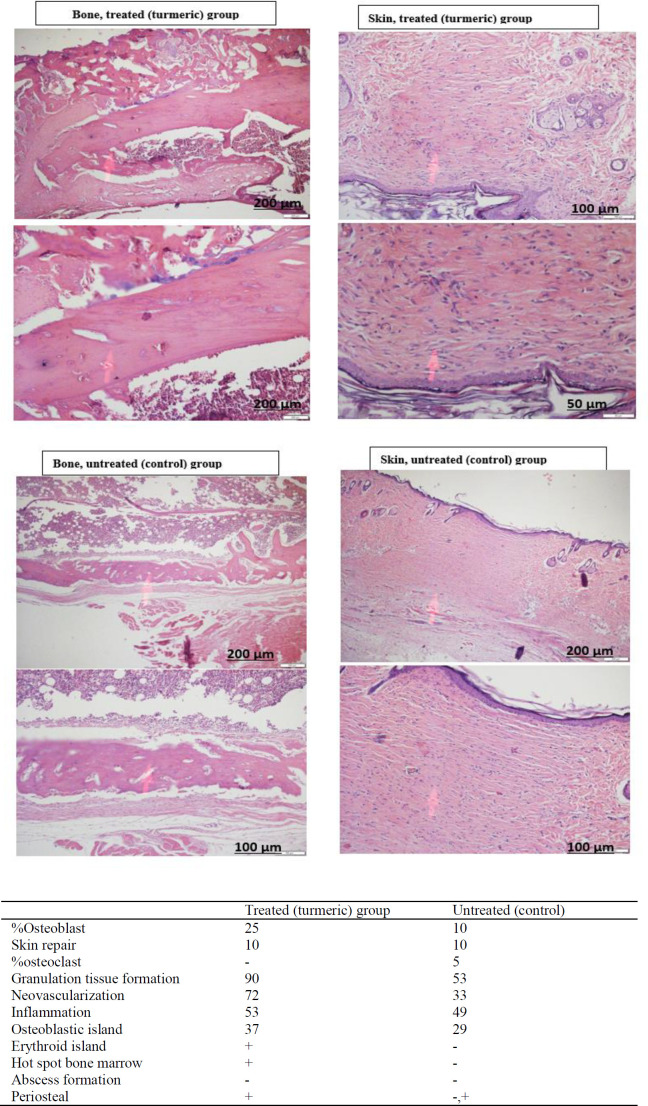
histologic sections of femur fracture site six weeks post-surgery in turmeric-treated and untreated animals. After six weeks following up the fracture induced animals, the percentage of osteoblasts was higher in in turmeric-treated group (25% vs 10%), whereas osteoclasts percentage was higher in control group (5% vs 0%). Moreover, tissue granulation and neovascularization were notably higher in treated animals. Cellular infiltration was observed in both groups. No abscess was formed in any studied animals.

**Table 1:** RANKL, and OPG in turmeric-treated animals in the femoral fracture-induced rat model

**Name**	**GAPDH**		**OPG**		**RANK**		**RANKL**		**OPG**	**RANK**	**RANKL**	**OPG**	**RANK**	**RANKL**	**RANKL/OPG**
	**Take Off**	**Ampl.**	**Take Off**	**Ampl.**	**Take Off**	**Ampl.**	**Take Off**	**Ampl.**	**∆CT**	**∆CT**	**∆CT**	**2^-∆CT**	**2^-∆CT**	**2^-∆CT**	
1	20.40	1.92	29.30	1.73	31.10	1.76	24.90	1.68	8.90	10.70	4.5	0.0021	0.00060	0.0442	21.1121
1	20.20	1.68	29.60	1.85	30.60	1.45	27.10	1.74	9.40	10.40	6.9	0.0015	0.00074	0.0084	5.6569
1	20.30	1.80	29.45	1.79	30.85	1.61	26.00	1.71	9.15	10.55	5.7	0.0018	0.00067	0.0192	10.9283
2	17.60	1.71	27.60	1.82	29.30	1.60	25.50	1.74	10.00	11.70	7.9	0.0010	0.00030	0.0042	4.2871
2	17.10	1.38	27.90	1.75	28.70	1.74	26.50	1.81	10.80	11.60	9.4	0.0006	0.00032	0.0015	2.6390
2	17.35	1.55	27.75	1.79	29.00	1.67	26.00	1.78	10.40	11.65	8.7	0.0007	0.00031	0.0025	3.3636
3	14.70	1.76	25.80	1.70	27.90	1.65	24.70	1.76	11.10	13.20	10.0	0.0005	0.00011	0.0010	2.1435
3	14.90	1.76	25.70	1.69	27.70	1.74	24.30	1.47	10.80	12.80	9.4	0.0006	0.00014	0.0015	2.6390
3	14.80	1.76	25.75	1.70	27.80	1.70	24.50	1.62	10.95	13.00	9.7	0.0005	0.00012	0.0012	2.3784
4	19.20	1.79	27.70	1.77	29.70	1.72	26.40	1.72	8.50	10.50	7.2	0.0028	0.00069	0.0068	2.4623
4	19.40	1.80	28.00	1.73	29.70	1.72	22.80	1.74	8.60	10.30	3.4	0.0026	0.00079	0.0947	36.7583
4	19.30	1.80	27.85	1.75	29.70	1.72	24.60	1.73	8.55	10.40	5.3	0.0027	0.00074	0.0254	9.5137

Ampl.: Amplification

**Table 2:** Relative expression of RANK, RANKL, and OPG in untreated animals in the femoral fracture-induced rat model

**Name**	**GAPDH**			**OPG**		**RANK**		**RANKL**		**OPG**	**RANK**	**RANKL**	**OPG**	**RANK**	**RANKL**	**RANKL/OPG**
	**Take Off**	**Ampl. **		**Take Off**	**Ampl.**	**Take Off**	**Ampl.**	**Take Off**	**Ampl.**	**∆CT**	**∆CT**	**∆CT**	**2^-∆CT**	**2^-∆CT**	**2^-∆CT**	
5	16.70	1.74	21.70		1.68	22.30	1.64	17.10	1.67	5.00	5.60	0.40	0.03125	0.0206	0.7579	24.2515
5	16.60	1.65	21.70		1.86	22.80	1.83	17.40	1.78	5.10	6.20	0.80	0.02916	0.0136	0.5743	19.6983
5	16.65	1.70	21.70		1.77	22.70	1.77	17.70	1.77	5.05	6.05	1.05	0.03019	0.0151	0.4830	16.0000
6	17.50	1.66	21.60		1.75	22.70	1.60	17.70	1.51	4.10	5.20	0.20	0.05831	0.0272	0.8706	14.9285
6	17.80	1.77	21.70		1.64	22.70	1.75	18.00	1.87	3.90	4.90	0.20	0.06699	0.0335	0.8706	12.9960
6	17.65	1.62	21.65		1.70	22.65	1.70	17.65	1.70	4.00	5.00	0.00	0.06250	0.0313	1.0000	16.0000
7	17.80	1.87	21.60		1.60	22.20	1.65	17.80	1.76	3.80	4.40	0.00	0.07179	0.0474	1.0000	13.9288
7	17.70	1.72	21.70		1.53	22.20	1.71	17.50	1.73	4.00	4.50	-0.20	0.06250	0.0442	1.1487	18.3792
7	17.75	1.80	21.65		1.57	21.65	1.57	17.65	1.57	3.90	3.90	-0.10	0.06699	0.0670	1.0718	16.0000
8	17.50	1.74	21.90		1.98	23.00	1.79	17.60	1.65	4.40	5.50	0.10	0.04737	0.0221	0.9330	19.6983
8	17.50	1.66	21.90		2.02	22.80	1.75	17.50	1.78	4.40	5.30	0.00	0.04737	0.0254	1.0000	21.1121
8	17.50	1.70	21.90		2.00	22.90	1.90	17.90	1.80	4.40	5.40	0.40	0.04737	0.0237	0.7579	16.0000

Ampl.: Amplification

**Table 3:** Relative expression of RANK, RANKL, and OPG in healthy control group

	**GAPDH**		**OPG**		**RANK**		**RANKL**		**OPG**	**RANK**	**RANKL**	**OPG**	**RANK**	**RANKL**	**RANKL/OPG**
**Name**	**Take Off**	**Ampl.**	**Take Off**	**Ampl.**	**Take Off**	**Ampl.**	**Take Off**	**Ampl.**	**∆CT**	**∆CT**	**∆CT**	**2^-∆CT**	**2^-∆CT**	**2^-∆CT**	
9	17.30	1.71	21.60	1.69	22.70	1.73	17.80	1.68	4.30	5.40	0.50	0.0508	0.0237	0.7071	13.9288
9	17.40	1.77	21.80	1.79	22.70	1.86	18.00	1.94	4.40	5.30	0.60	0.0474	0.0254	0.6598	13.9288
9	17.35	1.54	21.70	1.59	22.70	1.75	17.90	1.81	4.35	5.35	0.55	0.0490	0.0245	0.6830	13.9288
10	17.90	1.70	21.60	1.45	22.90	1.68	17.40	1.73	3.70	5.00	-0.50	0.0769	0.0313	1.4142	18.3792
10	17.20	1.59	21.70	2.17	21.10	1.53	17.40	1.75	4.50	3.90	0.20	0.0442	0.0670	0.8706	19.6983
10	17.55	1.65	21.65	1.81	22.00	1.61	17.40	1.54	4.10	4.45	-0.15	0.0583	0.0458	1.1096	19.0273
11	17.20	1.32	21.90	2.06	22.70	1.73	17.80	1.68	4.70	5.50	0.60	0.0385	0.0221	0.6598	17.1484
11	17.80	1.64	22.20	1.83	23.00	1.64	17.80	1.81	4.40	5.20	0.00	0.0474	0.0272	1.0000	21.1121
11	17.50	1.68	22.05	1.95	22.85	1.69	17.80	1.75	4.55	5.35	0.30	0.0427	0.0245	0.8123	19.0273
12	17.80	1.77	21.80	1.68	22.90	1.63	17.70	1.79	4.00	5.10	-0.10	0.0625	0.0292	1.0718	17.1484
12	17.50	1.78	21.50	1.73	23.20	1.69	17.90	1.74	4.00	5.70	0.40	0.0625	0.0192	0.7579	12.1257
12	17.65	1.78	21.65	1.71	23.05	1.56	17.80	1.77	4.00	5.40	0.15	0.0625	0.0237	0.9013	14.4200

Ampl.: Amplification
